# Gender disparity in health-related quality of life among people living with HIV/AIDS in Ethiopia: a systematic review and meta-analysis

**DOI:** 10.3389/fgwh.2024.1471316

**Published:** 2024-11-20

**Authors:** Derara Girma Tufa, Habteyes Hailu Tola, Hiwot Dejene Dissassa, Leta Adugna Geleta, Erean Shigign Malka, Addisu Waleligne Tadesse, Feyiso Bati Wariso, Getahun Fetensa

**Affiliations:** ^1^ Public Health Department, College of Health Sciences, Salale University, Fiche, Ethiopia; ^2^ Department of Public Health, College of Health Sciences, Dire Dawa University, Dire Dawa, Ethiopia; ^3^ Department of Health Behavior and Society, Faculty of Public Health, Institute of Health, Jimma Medical Center, Jimma University, Jimma, Ethiopia; ^4^ Department of Nursing, Institute of Health Sciences, Wollega University, Nekemte, Ethiopia; ^5^ Center for Evidence-Synthesis, Support, and Development in Africa (CESDA), PLC, Addis Ababa, Ethiopia

**Keywords:** health-related quality of life, gender disparity, HIV/AIDS, meta-analysis, Ethiopia

## Abstract

**Introduction:**

Health-related quality of life (HRQoL) is a key outcome indicator in antiretroviral therapy program. In Ethiopia, primary studies on gender disparity in HRQoL among people living with HIV/AIDS (PLHA) are conflicting, with no pooled estimation. Therefore, this study aimed to investigate gender disparity in HRQoL among PLHA in Ethiopia.

**Methods:**

Studies were retrieved from PubMed, Web of Science, SCOPUS, Embase, MEDLINE, Science Direct, HINARI, and PsycINFO were systematically searched. In addition, Google Scholar, Google, journal homepages, bibliographies, and universities' research repositories in the country were searched by combining keywords and Medical Subject Headings (MeSH) terms with Boolean operators. Based on the primary study results, the average score of each domain was utilized as a cut-off point to classify HRQoL as poor or good. The Joanna Briggs Institute (JBI) checklist was used to assess study quality. A random-effects model was used to report the pooled estimates. Summary estimates are presented in forest plots and tables. The variation between studies was assessed using the Higgins heterogeneity test (I^2^). Funnel plot, Begg's test, and Egger's test were used to assess publication bias. Data were extracted using Microsoft Excel and exported to STATA 17 (Corporation, College Station, TX, USA) for analysis. The search results were managed using the EndNote X7 software.

**Results:**

Fifteen studies with 4,867 PLHA were included. The pooled prevalence of poor HRQoL was 46.53% (95% CI: 41.96–51.10), 46.15% (95% CI: 37.05–55.24), and 36.21% (95% CI: 30.19–42.23) among PLHA, women, and men living with HIV, respectively. Moreover, a significant gender disparity in HRQoL was observed among Ethiopian women and men living with HIV. Women living with HIV were found to have 61% increased odds of poor HRQoL than men living with HIV in the country (OR = 1.61, 95% CI: 1.07, 2.15). No statistical evidence of a publication bias was observed.

**Conclusion:**

Almost half of PLHA patients in Ethiopia had a poor HRQoL. Women living with HIV have higher odds of poor HRQoL than men living with HIV. This highlights the pressing need for gender-specific risk assessment approaches and treatment interventions aimed at optimizing HRQoL in HIV/AIDS settings.

**Systematic Review Registration:**

https://www.crd.york.ac.uk/prospero/, identifier CRD42023454810.

## Introduction

Human Immunodeficiency Virus (HIV) infection remains a major global public health problem. Since its occurrence, 85.6 million [65.0–113.0] individuals have been infected, and about 40.4 million [32.9–51.3] have died. By the end of 2022, 39.0 million [33.1–45.7] individuals were living with HIV worldwide. World Health Organization (WHO) African region continues to be the most seriously affected, accounting for more than two-thirds of all people living with HIV/Acquired Immuno-deficiency Virus (AIDS) (PLHA) globally (1). Specifically, HIV is a major public concern in Ethiopia, with an estimated prevalence of 1.16% in the adult population (2).

Women are disproportionately affected by HIV/AIDS (3) accounting for 53% of all PLHA (1). Gender inequality and disparities have increased the epidemic, resulting in the violation of women's reproductive rights (4, 5). Unequal power relations, sexual coercion, and violence are common experiences for women of all ages, and they have various detrimental effects on female sexual, physical, and mental health, predisposing them to HIV infection (5). Globally, HIV/AIDS infection demonstrates the destructive repercussions of gender discrimination on human health and the socioeconomic structure of society (5).

The WHO defines quality of life (QoL) as an individual's view of their position in life within the context of the culture and value systems in which they live, as well as their goals, expectations, standards, and concerns (6). Health-related quality of life (HRQoL) refers to QoL in the context of health, disease, and treatment (7). It also denotes patient reports on functioning and well-being in the physical, mental, and social dimensions of life (8). As a result, assessing HRQoL can provide an accurate assessment of how patients' lives are affected by disease and treatment (9, 10). Besides, for those living with chronic diseases, HRQoL is an important healthcare metric (11). Subsequently, among PLHA, the poor HRQoL was often defined as a score below the average in each domain and their overall summation (12–19).

HIV is increasingly seen as a chronic disease that affects not only physical health but also the mental and social conditions of patients, due to the negative attitude of society, discrimination, and stigma, especially in developing countries (20). Evidence suggests that, in addition to underlying infection, social circumstances, relationship issues, comorbidities, and stigma affect the HRQoL of PLHA (21). Thus, HRQoL is described as one of the key factors in evaluating the health status of PLHA, and its improvement is an important treatment goal (22). In 2016, a 4th 90 target was introduced to enhance the HRQoL for PLHA by addressing comorbidities and psychosocial issues, alongside the existing triple 90 targets for clinical health monitoring. Specifically, this 4th target aims to improve the HIV care continuum in sub-Saharan Africa (SSA) by focusing on various factors to enhance HRQoL among PLHA (23).

HRQoL among PLHA is frequently assessed by the WHO Quality-of-Life Bref (QoL-HIV BREF) in six domains: physical, social relationships, level of independence, spiritual, psychological, and environmental (22, 24). The other most commonly used instruments are the Medical Outcome Survey-HIV (MOS-HIV), Short Form-36 (SF-36), European Quality-of-Life Instrument-5 Dimension (EQ-5D), Short Form-12 (SF-12), and HIV/AIDS Targeted Quality-of-Life (HAT-QOL). Furthermore, it has been shown that there is no significant variation in completion rates between the different HRQoL tools (24). Moreover, different studies have utilized below-average scores on these tools to categorize HRQoL as poor and otherwise as good status (12–15, 25).

Studies on gender disparity in HRQoL among PLHA have shown inconsistent results. Some studies have reported no disparity (16, 26, 27). However, others have suggested that there is an important gender disparity in HRQoL. Subsequently, several studies have shown that women had significantly lower scores compared to men (28–31). In contrast, a limited finding stated that women had higher HRQoL domain scores than men (32). Overall, establishing gender disparity in HRQoL can influence policies on integrating gender equality in HIV care services as an approach for improving the lives of PLHA (28).

In Ethiopia, 45.27% of PLHA had poor HRQoL (33). However, no pooled study has been conducted to identify gender disparity in HRQoL. Furthermore, different primary studies conducted in the country showed conflicting findings on the gender disparity in HRQoL (12–14, 16, 17, 25–27, 29, 31, 34). Therefore, this systematic review and meta-analysis (SRMA) aimed to estimate the pooled prevalence of poor HRQoL and gender disparity among PLHA in Ethiopia.

## Methods

### Protocol registration and reporting

The protocol for this study was registered in the PROSPERO with the registration number CRD42023454810 and link https://www.crd.york.ac.uk/prospero/. The Preferred Reporting Items for Systematic Reviews and Meta-Analysis (PRISMA) guidelines were used to report the results of this systematic review and meta-analysis (35) (Supplementary File 1).

### Literature search

To conduct this SRMA, relevant articles and grey literature were searched. PubMed, Web of Science, SCOPUS, Embase, MEDLINE, Science Direct, HINARI, and PsycINFO were systematically searched. Studies were also retrieved from Google Scholar, Google, journal homepages, bibliographies, and university research repositories in the country. Keywords: “HIV”, “human immunodeficiency virus”, “acquired immunodeficiency syndrome”, “AIDS”, “HIV Infection”, “HIV positive”, “people living with HIV/AIDS”, “HIV/AIDS”, “antiretroviral therapy”, “ART”, “highly active anti-retroviral therapy”, “HAART”, “Health-related quality of life”, “Quality of Life”, “HRQOL”, “QOL”, “Ethiopia” were used in free text and MeSH terms. The search results were managed using EndNote X7 software.

### Eligibility criteria

This SRMA included studies conducted in Ethiopia on all adult PLHA that were accessible before November 17, 2023. Original full-text articles and studies written in English were included. Studies that did not report the prevalence of poor HRQoL and did not report on both genders were excluded.

### Review questions

• What is the pooled prevalence of poor HRQoL among PLHA in Ethiopia?
• Is there a gender disparity in HRQoL among PLHA in Ethiopia?

### Outcome measurements

The prevalence of poor HRQoL, as determined by any of the standardized instruments used in the original studies, was considered. Based on the primary study results, the average score of each domain was utilized as a cut-off point to classify HRQoL as poor or good. Likewise, the same score was used to categorize the domains as poor or good HRQoL. Furthermore, the gender disparity in HRQoL was assessed by identifying studies reporting the association between gender and HRQoL and aggregating them using odds ratios (OR).

### Data extraction

Data from each study were extracted by four independent authors (DGT, HDD, ESM, and AWT) in a customized format in Microsoft Excel. Inconsistencies between the authors have been resolved by other authors. The extracted data included author names, year of publication, sample size, outcome, study design, region, and settings in which the studies were conducted (Supplementary File 2).

### Quality and risk of bias assessment

The Joanna Briggs Institute (JBI) tools for cross-sectional and case-control studies (36) were used to assess the methodological quality and risk of bias (in design, conduct, and analysis) of the included studies (Supplementary File 3). The tools were used to evaluate the inclusion criteria, measurement of outcome variables, confounding adjustment, and appropriateness of statistical analysis. The quality of the extracted studies was evaluated by all authors who participated in data extraction.

### Data analysis

STATA 17 (Corporation, College Station, TX, USA) was used for data analysis. The pooled prevalence of poor HRQoL was determined. To detect the gender disparity in poor HRQoL, the pooled results were presented as odds ratios (ORs) with 95% confidence intervals (CI). Heterogeneity among the included studies was checked using Cochran's Q (χ^2^ test) and I^2^ test. The heterogeneity was considered as low, moderate or high when I^2^ test statistics results were 25%, 50%, and 75% respectively (37). Forest plots were used to visualize heterogeneity. Because of heterogeneity, a random-effects model was used to estimate the Der Simonian and Laird's pooled effect. Subgroup and meta-regression analyses were performed to identify the sources of heterogeneity. Publication bias was assessed by using a funnel plot of symmetry. Furthermore, the statistical significance of publication bias was checked using the Egger and Begg tests (38). A *p*-value less than 0.05 was used to declare the presence of publication bias. Sensitivity analysis using a random-effects model was performed to assess the influence of a single study on the overall meta-analysis estimate.

## Results

### Identification and selection of studies

**Figure 1:**
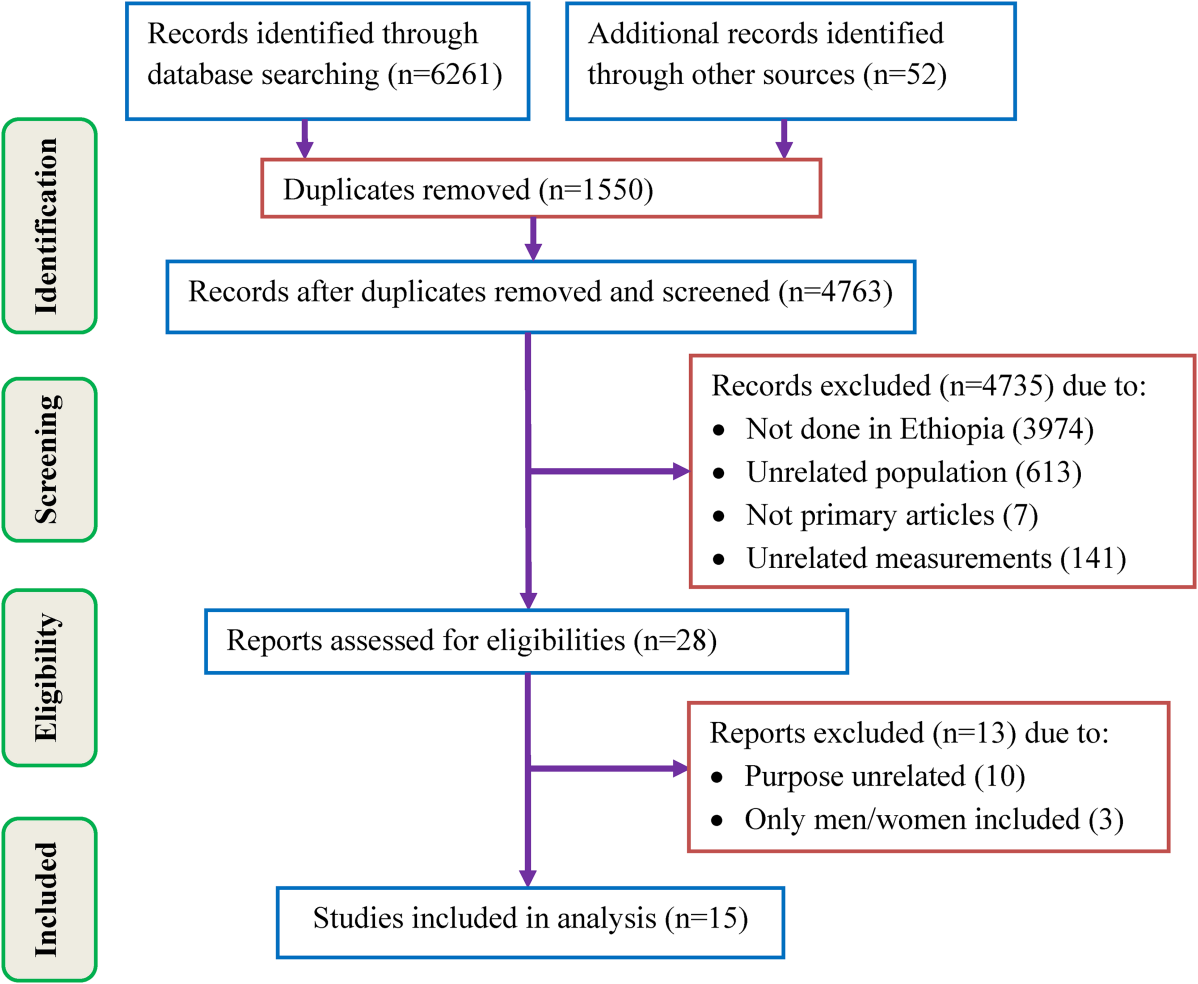
PRISMA 2020 flow diagram showing the selection process of studies.

Initially, 6,313 studies were retrieved by searching databases and other sources, followed by the exclusion of 1,550 studies because of duplication. Of the remaining 4,763 studies, 4,735 were removed by title and abstract screening due to different reasons including, not done in Ethiopia (3,974), unrelated population (613), not primary articles (7), and unrelated measurements (141). Then, 28 studies were eligible for the full assessment. Of these, 10 and 3 studies were removed due to unrelated purposes and did not include both genders, respectively. Finally, 15 studies that met the inclusion criteria were included in the final SRMA (Figure 1).

### Study characteristics

This SRMA included 15 original studies with a total pooled sample size of 4,867 PLHA, of which 2,857 (58.7%) were women. All studies were published articles (12–19, 25–27, 39–41), except one unpublished article (34). Furthermore, 14 studies used a cross-sectional study design (12–16, 18, 19, 25–27, 34, 39–41) and one study used a case-control study design (17). The minimum sample size was 95 (13), and the maximum was 428 (12). Six studies were conducted in the Amhara region (12, 16, 18, 34, 39, 40), five in Oromia (13–15, 17, 27), two in Benishangul-Gumuz (25, 41), one in Southwest Ethiopia (26), and one in Southern Ethiopia (19). Besides, ten studies used the WHO QOL-HIV-BREF (12–19, 34), two studies used the MOS HIV questionnaire (40, 41), one study used PROMIS-GMH (25) to measure HRQoL, and the tool was not reported in 2 studies (27, 39) (Table 1).

**Table 1:** General characteristics of included studies.

Authors	Year	Sample size	RR (%)	Total female	Total male	Age group	Study design	Sampling technique	Region	Study area	Tool used
Askal et al.	2011	394	100	239	155	≥19	Cross-sectional	Systematic RS	Amhara	Wollo	WHO QOL-HIV-BREF
Alemu et al.	2013	424	100	266	158	NR	Cross-sectional	Systematic RS	Amhara	Bahir Dar	NR
Surur et al.	2017	400	85.47	219	181	>18	Cross-sectional	Systematic RS	Amhara	Gondar	WHO QOL-HIV-BREF
Ayalew et al.	2018	300	100	128	172	≥18	Cross-sectional	Simple RS	Amhara	Gondar	WHO QOL-HIV-BREF
Tesfaye et al.	2018	351	94.35	232	119	NR	Cross-sectional	Systematic RS	Oromia	Jimma	NR
Paulos et al.	2019	300	96.5	155	145	>18	Cross-sectional	Systematic RS	Benishangul Gumuz	Asosa	MOS HIV questionnaire
Zeleke et al.	2019	95	97.90	56	39	NR	Cross-sectional	Census	Oromia	Addis Ababa & Jimma	WHO QOL-HIV-BREF
Legesse et al.	2019	391	94	232	159	≥15	Cross-sectional	Systematic RS	South Ethiopia	Arbaminch	WHO QOL-HIV-BREF
Desta et al.	2020	240	77.2	153	87	≥18	Cross-sectional	Consecutive	Southwest Ethiopia	Mizan-Aman	WHO QOL-HIV-BREF
Dinsa et al.	2020	296	87.06	168	128	>18	Cross-sectional	Simple RS	Oromia	Ambo	WHO QOL-HIV-BREF
Gesese	2021	323	100	199	124	≥15	Case-control	Census/simple RS	Oromia	Jimma	WHO QOL-HIV-BREF
Yohannes et al.	2021	428	96.6	228	200	≥18	Cross-sectional	Systematic RS	Amhara	Debre Markos	WHO QOL-HIV-BREF
Mohammed et al.	2021	235	93.62	136	99	≥18	Cross-sectional	Systematic RS	Amhara	Dessie	MOS HIV questionnaire
Nigusso et al.	2021	390	98.98	259	131	≥18	Cross-sectional	Simple RS	Benishangul Gumuz	Benishangul Gumuz	PROMIS-GMH
Gesese et al.	2022	300	97.7	187	113	≥15	Cross-sectional	Simple RS	Oromia	Jimma	WHO QOL-HIV-BREF

NR, not reported; RS, random sampling.

### Prevalence of poor HRQoL among PLHA

**Figure 2:**
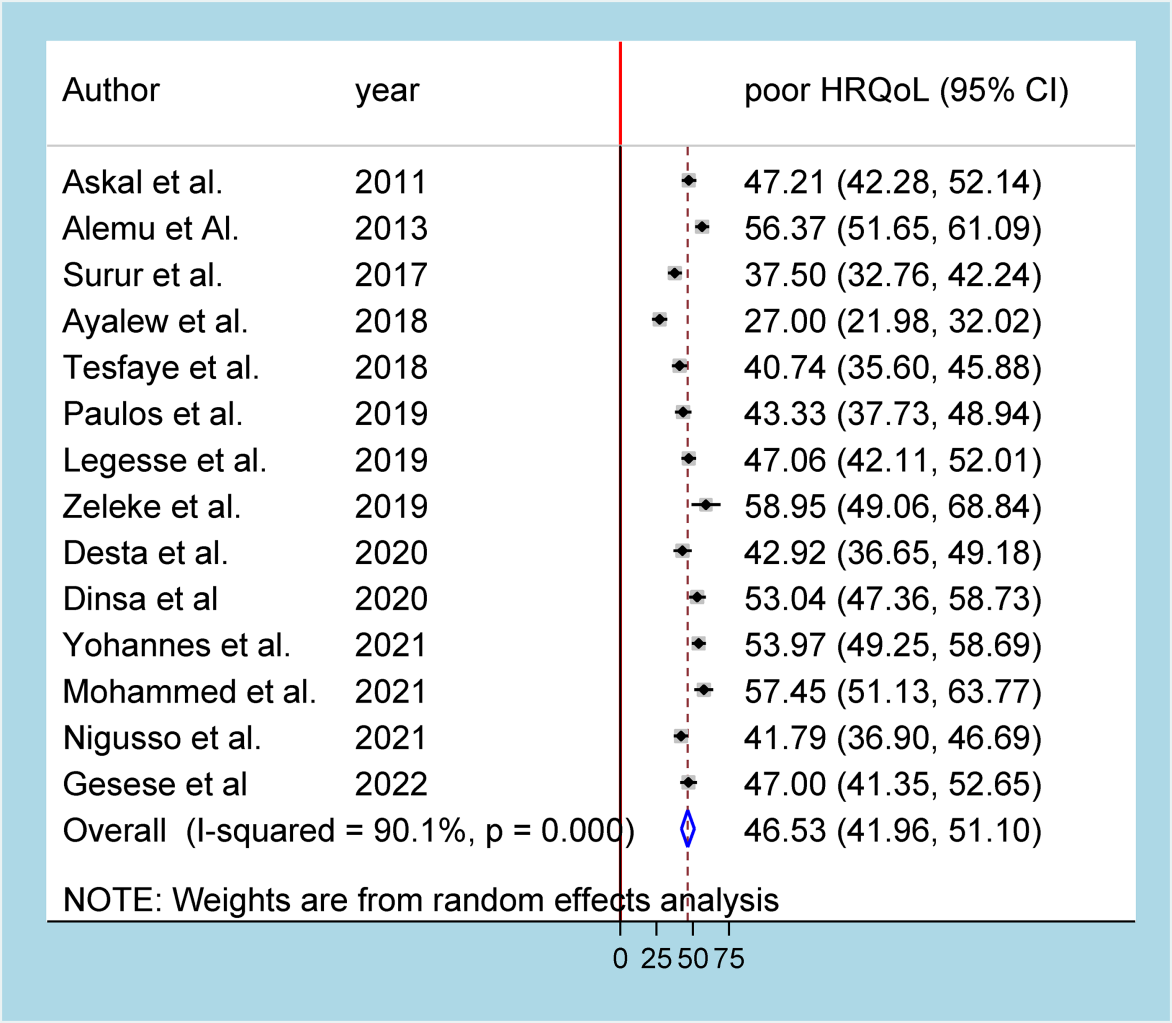
Forest plot showing the pooled prevalence of HRQoL among PLHA in Ethiopia.

In the meta-analysis of 14 studies that reported on the prevalence of poor HRQoL, there was high heterogeneity (I^2^ = 90.1%, *p*-value = 0.000), and thus, a random effects model was used to analyze the results. As a result, the pooled prevalence of poor HRQoL was found to be 46.53% (95% CI: 41.96–51.10) (Figure 2). Moreover, the pooled prevalence of poor HRQoL among women and men living with HIV in Ethiopia was 44.46% (95% CI: 35.46–53.47) and 41.52% (95% CI: 29.89–53.15), respectively (Supplementary File 2).

**Figure 3:**
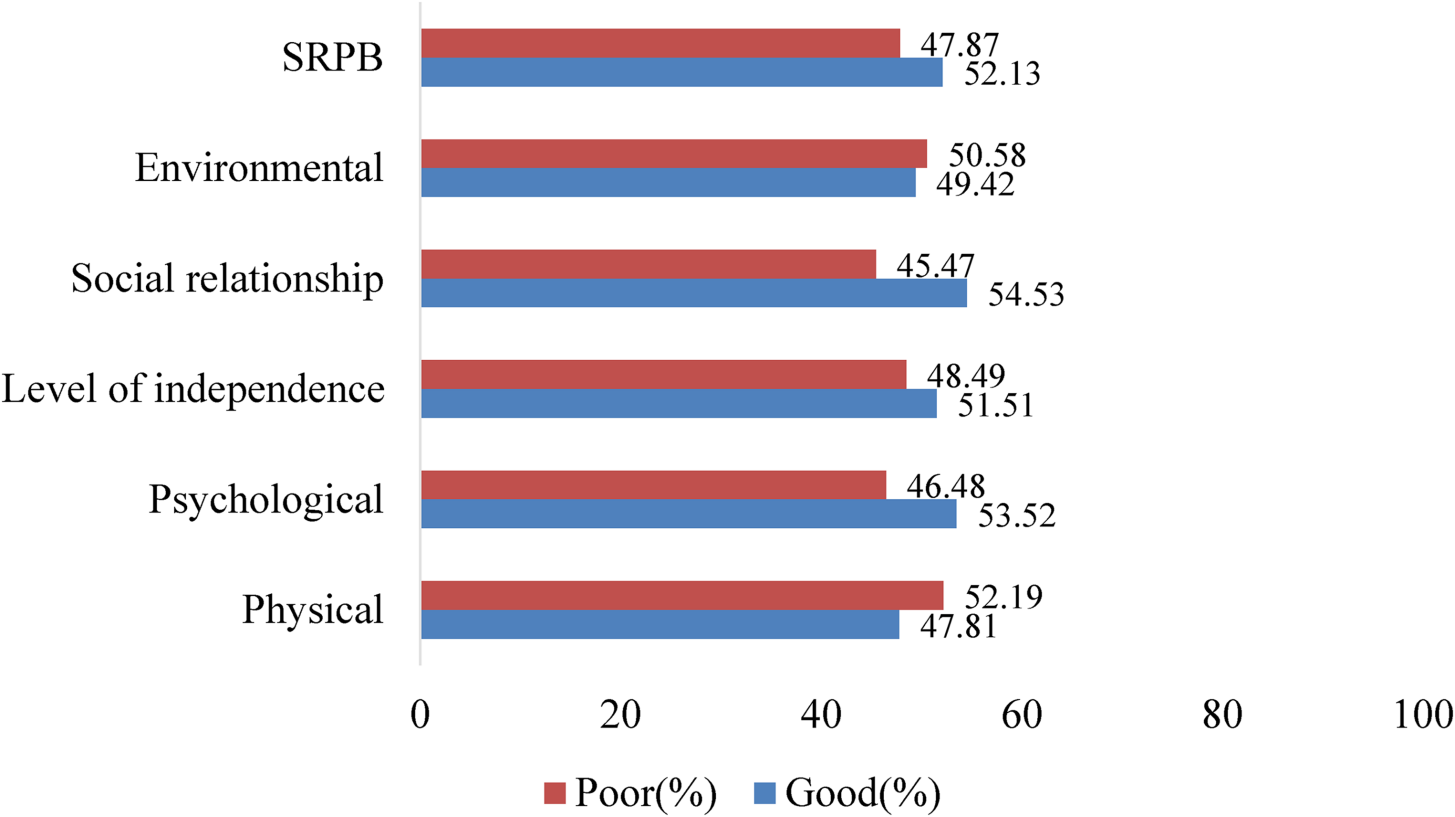
Pooled prevalence of the six HRQoL domains for PLHA using the wHO QOL-HIV-BREF tool in Ethiopia.

Furthermore, the pooled estimation of the PLHA's HRQoL indicates that the domains with the highest prevalence of poor HRQoL were physical (52.19%) and environmental (50.58%). In contrast, the social relationship was identified as the domain with the highest score of good HRQoL (54.53%) (Figure 3).

### Gender disparity in poor HRQoL among PLHA

**Figure 4:**
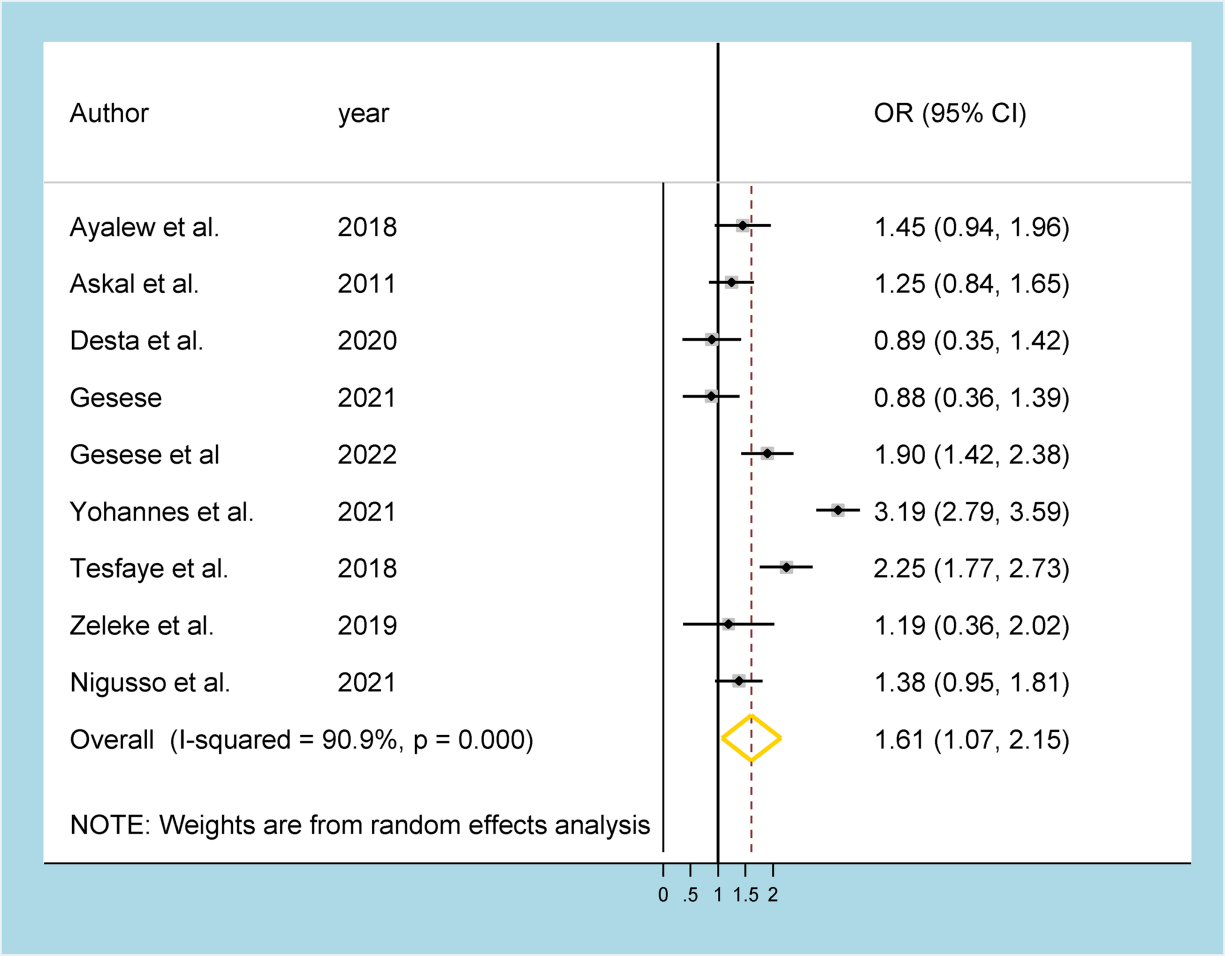
Forest plot showing the gender disparity among PLHA in Ethiopia.

Of the included studies, nine studies (12–14, 16, 17, 25–27, 34) were reported on the association between gender and HRQoL. Subsequently, significant gender disparity was observed among Ethiopian women and men living with HIV/AIDS in HRQoL using the random-effects model (I^2^ = 90.9%, *p*-value = 0.000). Subsequently, women living with HIV were found to have 61% increased odds of poor HRQoL than men living with HIV in the country (OR = 1.61, 95% CI: 1.07, 2.15) (Figure 4).

### Sub-group analysis and meta-regression

Furthermore, the sources of heterogeneity were investigated using sub-group analysis and meta-regression techniques. The Sub-group analysis was done by regions. Accordingly, a significant heterogeneity was observed in Oromia and Amhara regions, while no significant heterogeneity was observed in other regions (South Ethiopia and Benishangul Gumz) (Supplementary File 4). Besides, meta-regression was performed considering the year of publication and the sample size of the studies. None of the variables were statistically significant for explaining the presence of heterogeneity. Accordingly, the meta-regression results revealed that heterogeneity in the gender disparity was unrelated to sample size variation (coefficient = 0.0042466, *p*-value = 0.166) or publication year (coefficient = 0.0631991, *p*-value = 0.437).

### Publication bias

The presence of publication bias was assessed using funnel plot and Egger, and Begg statistical tests. In funnel plot, visual inspection showed that the presence of publication bias was less likely as there was relatively symmetrical study distribution of the study in the graph (Supplementary File 4). Also, there was no statistical evidence of publication bias in the pooled estimates using the Egger and Begg tests (*p*-value = 0.276 and *p*-value = 0.211), respectively.

### Sensitivity analysis

To check the individual effect of included studies on the gender disparity in HRQoL among PLHA in Ethiopia, sensitivity analysis was done using random effect model. The findings revealed that there was no strong evidence that a single study had an impact on the pooled result of the meta-analysis, as the single study estimates were closer to the combined estimate (Supplementary File 4).

## Discussion

This SRMA estimated the prevalence and gender disparity in poor HRQoL among PLHA in Ethiopia. Thus, the pooled prevalence of poor HRQoL was 46.53% (95% CI: 41.96–51.10). A previous study from Ethiopia reported a comparable result (45.27%) (33). This might be due to the similarity in the study settings, populations, and the same articles included in both studies. Continued patterns of poor HRQoL among PLHA in the country highlight the need to recommend and implement the additional 4th UNAIDS target, ensuring 95% of PLHA experience the best possible HRQoL (42).

In contrast to the current study, the studies from other African countries revealed considerably a lower prevalence of poor HRQoL, 23.8% from Togo (43) and 21.6% from Botswana (7). The discrepancy in findings could be explained by differences in research designs and the age of the study participants. Age was depicted as having the inverse association with HRQoL in studies among PLHA (44–46). Furthermore, the need to investigate particular populations is stressed because sociodemographic variances can result in conclusions that contradict (47).

In this study, women living with HIV had significantly higher odds of poor HRQoL than men living with HIV. Various studies evinced this finding (30, 48). Different interpretations of the findings were postulated based on the domains of HRQoL. For instance, women appear to be more harmed in their physical HRQoL by personal rejection, experiencing social stigma from a variety of sources, including their communities, interpersonal encounters, and inside systems and organizations (49). Additionally, the stigma associated with HIV/AIDS could lower environment HRQoL among women who continued to be productive and the larger amount of performing chores (50). Women also reported more pain, less energy, and poor physical and mental health HRQoL (51). Furthermore, women living with HIV have a lower source of income, engage in less economic activities for their families, and have a greater burden in duties such as cooking and childcare than men (52). Cumulatively, insights into any of the dimensions could direct interventions used to improve HRQoL among PLHA taking into account gender disparity.

The current disparity in HRQoL is also confirmed by the studies demonstrating that HRQoL among older PLHA differs significantly by gender, implying high-priority interventions to reduce gender disparities in HRQoL and improve the structural conditions in which they live (53). Also, using the gender-specific HRQoL parameters in program development is suggested to improve the cost-effectiveness of interventions (54). Another study also revealed a considerably higher score of poor HRQoL among women living with HIV and recommends improving social and economic status and establishing a women's social network for women living with HIV (55).

In particular, primary studies conducted in different places in Ethiopia also reported significantly a higher risk of experiencing poor HRQoL among women living with HIV than men living with HIV (12, 29, 31, 52). This demonstrates that women living with HIV have consistently a higher prevalence of poor HRQoL than men living with HIV in the country. The spotted gaps in gender advocate for more gender mainstreaming in HIV service delivery in the country (28). Understanding these gender disparities can give important information for customizing interventions to improve HRQoL among PLHA.

## Strengths and limitations

This is the first study in Ethiopia to examine gender disparity in HRQoL among PLHA. This finding broadens current research on gender disparity in HRQoL by offering reliable evidence from a large national sample of PLHA. However, the study did have several shortcomings. The majority of the research were done in the Oromia and Amhara areas, which may not be indicative of the country as a whole. Furthermore, because of using different HRQoL measurement tools, it was not possible to pool all dimensions used in generating HRQoL. Furthermore, this study used categorical data (odds ratios), not continuous data (β-coefficients) to analyze the relationship between gender and HRQoL.

## Conclusion

This study revealed that nearly half of PLHA in Ethiopia had poor HRQoL, showing that it is a persistent significant public health concern in this population. Moreover, a significant gender disparity in HRQoL has been observed among PLHA. Women were more likely than men to have poor HRQoL. Therefore, this highlights the pressing need for gender-specific risk assessment approaches and treatment interventions aimed at optimizing HRQoL in the HIV/AIDS setting. Moreover, the establishment of relevant measures to track progress towards improving HRQoL of PLHA should prioritize women as part of the action plan in Ethiopia. Furthermore, this finding suggests carrying out further studies to identify the key bio-psycho-social variables driving gender disparities in HRQoL among PLHA.

## Data Availability

The original contributions presented in the study are included in the article/Supplementary Material, further inquiries can be directed to the corresponding author.
