# Supplementary material for: Gender disparity in health-related quality of life among people living with HIV/AIDS in Ethiopia: a systematic review and meta-analysis
Source: Front Glob Womens Health. 2024 Nov 20;5:1471316. doi: 10.3389/fgwh.2024.1471316 (PMC11614810; doi:10.3389/fgwh.2024.1471316)
Supplement: Supplementary file 2 [file Table3.docx]

| Author (year) | Were the criteria for inclusion in the sample clearly defined? | Were the study subjects and the setting described in detail? | Was the exposure measured in a valid and reliable way? | Were objective, standard criteria used for measurement of the condition? | Were confounding factors identified? | Were strategies to deal with confounding factors stated? | Were the outcomes measured in a valid and reliable way? | Was appropriate statistical analysis used? |
| --- | --- | --- | --- | --- | --- | --- | --- | --- |
| Alemu et Al. | Unclear | Yes | Yes | Yes | Yes | Yes | Unclear | Yes |
| Ayalew et al. | Yes | Yes | Yes | Yes | Yes | Yes | Yes | Yes |
| Askal et al. | Yes | Yes | Yes | Yes | Yes | Yes | Yes | Yes |
| Desta et al. | Yes | Yes | Yes | Yes | Yes | Yes | Yes | Yes |
| Dinsa et al | Yes | Yes | Yes | Yes | Yes | Yes | Yes | Yes |
| Gesese | Yes | Yes | Yes | Yes | Yes | Yes | Yes | Yes |
| Gesese et al | Yes | Yes | Yes | Yes | Yes | Yes | Yes | Yes |
| Paulos et al. | Yes | Yes | Yes | Yes | Yes | Yes | Yes | Yes |
| Yohannes et al. | Yes | Yes | Yes | Yes | Yes | Yes | Yes | Yes |
| Legesse et al. | Yes | Yes | Yes | Yes | Yes | Yes | Yes | Yes |
| Mohammed et al. | Yes | Yes | Yes | Yes | Yes | Yes | Yes | Yes |
| Tesfaye et al. | Unclear | Yes | Yes | Yes | Yes | Yes | Unclear | Yes |
| Zeleke et al. | Unclear | Yes | Yes | Yes | Yes | Yes | Yes | Yes |
| Nigusso et al. | Yes | Yes | Yes | Yes | Yes | Yes | Yes | Yes |
| Surur et al. | Yes | Yes | Yes | Yes | Yes | Yes | Yes | Yes |
