# Supplementary material for: Gender disparity in health-related quality of life among people living with HIV/AIDS in Ethiopia: a systematic review and meta-analysis
Source: Front Glob Womens Health. 2024 Nov 20;5:1471316. doi: 10.3389/fgwh.2024.1471316 (PMC11614810; doi:10.3389/fgwh.2024.1471316)
Supplement: Supplementary file 3 [file Table4.docx]

Figure 1: Forest plot of poor HRQoL among men living with HIV/AIDS

Figure 2: Forest plot of poor HRQoL among women living with HIV/AIDS

Figure 3: Subgroup analysis for the gender disparity


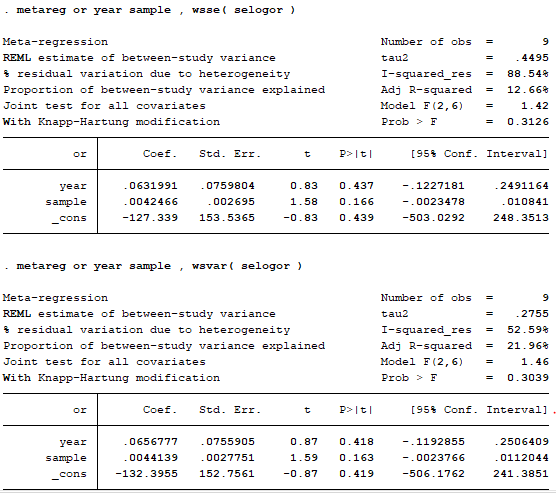


Figure 4: Meta-regression for gender disparity by sample size and year of publication

Figure 5: meta funnel depicting the symmetry of the studies


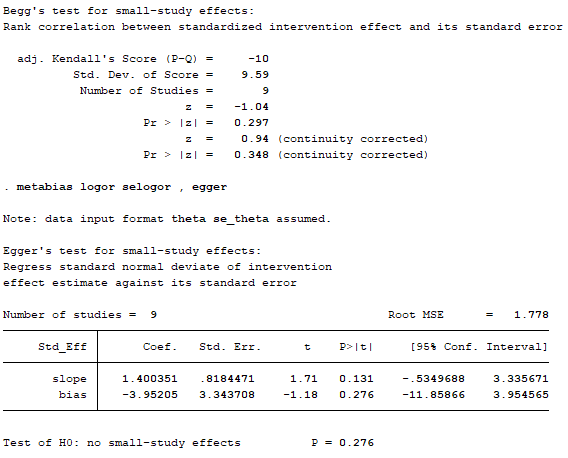


Figure 6: Beeg and egger test that indicate the absence of publication bias.

Figure 7: Sensitivity analysis
